# Dietary Patterns, Gut Microbiota Remodeling, and Cardiometabolic Disease

**DOI:** 10.3390/metabo13060760

**Published:** 2023-06-17

**Authors:** Letizia Guiducci, Giuseppina Nicolini, Francesca Forini

**Affiliations:** CNR Institute of Clinical Physiology, Via Moruzzi 1, 56124 Pisa, Italy; nicolini@ifc.cnr.it (G.N.); simona@ifc.cnr.it (F.F.)

**Keywords:** gut microbiota/heart axis, dysbiosis, cardiometabolic risk and disease, nutritional habits

## Abstract

The cardiovascular and metabolic disorders, collectively known as cardiometabolic disease (CMD), are high morbidity and mortality pathologies associated with lower quality of life and increasing health-care costs. The influence of the gut microbiota (GM) in dictating the interpersonal variability in CMD susceptibility, progression and treatment response is beginning to be deciphered, as is the mutualistic relation established between the GM and diet. In particular, dietary factors emerge as pivotal determinants shaping the architecture and function of resident microorganisms in the human gut. In turn, intestinal microbes influence the absorption, metabolism, and storage of ingested nutrients, with potentially profound effects on host physiology. Herein, we present an updated overview on major effects of dietary components on the GM, highlighting the beneficial and detrimental consequences of diet–microbiota crosstalk in the setting of CMD. We also discuss the promises and challenges of integrating microbiome data in dietary planning aimed at restraining CMD onset and progression with a more personalized nutritional approach.

## 1. Introduction

Cardiometabolic disorders continue to be the most significant and leading causes of morbidity and mortality worldwide. Though cardiovascular disease (CVD) have the greatest prevalence, closely related disorders, like diabetes mellitus and metabolic syndrome, greatly contribute to the overall public health burden. Environmental factors have a predominant role in CMD pathogenesis, and it has long been known that diet is a major modifiable contributor to the risk of developing CMD [1]. Accordingly, the last guidance on diet counseling introduced by the American Heart Association (AHA) to improve CV health emphasizes a whole foods approach, rather than focusing on a single nutrient, encouraging a higher intake of fresh vegetables, fruits, and whole grain rich in fibers and limiting red meat and the ultra-processed foods, as well as beverages and foods with added sugars. [2]. In recent years, GM has emerged as a critical link between diet and CMD [3]. Microbial communities residing in the intestinal tract represent a key endocrine organ able to establish a mutualistic relationship with the host [4]. On one hand, the GM has the enzymatic battery necessary to transform the ingested food through metabolic reactions that cannot be carried out by the host. Therefore, dietary factors are among the most potent modulators of microbiota composition and function. In turn, the microbially produced compounds function as metabolic substrates and signaling molecules, with major implications for host metabolism and health. A diet-driven GM dysbiosis can directly promote inflammatory pathways. Initially restricted to the intestine, this process favors the dysfunction or breakdown of the intestinal barrier, resulting in gut hyperpermeability and transfer of microorganisms and microorganism-derived toxins to the systemic circulation, a condition known as endotoxemia. GM adverse remodeling has been related to CMD, such as obesity, type 2 diabetes mellitus and CVD [5,6,7,8,9,10], thus, correcting the microbial gut imbalance through diet intervention, may offer a possibility to reduce cardiometabolic risk. It is noteworthy that the gut microbiome is estimated to exceed the number of genes in the human genome by two orders of magnitude. Such a diversity in composition, in addition to host genetic predisposition, plays a significant role in person to person variations in disease susceptibility, and in responses to diet [11]. As a consequence, personalized nutritional advices cannot ignore the basal GM composition. Technological advances in methods for GM profiling and other omic analytic platforms, such as metabolomic, can now be implemented not only to discover candidate microorganisms of relevance to CMD-related phenotypes, but also to understand the interaction between diet (input) and GM-derived metabolites (output) in order to guide better personalized nutritional plans. In this review, we present an overview of the latest findings on the impact of diet/GM crosstalk on CMD. First, the effects of macronutrients on GM function and composition is described with a focus on GM-derived compounds affecting CMD risk factors. The role of diet-driven gut dysbiosis in the onset and evolution of CMD is also discussed along with the potential of personalized dietary intervention aimed at re-establishing a healthier GM composition to prevent CMD progression.

## 2. Effects of Diet on GM Composition

**Figure 1:**
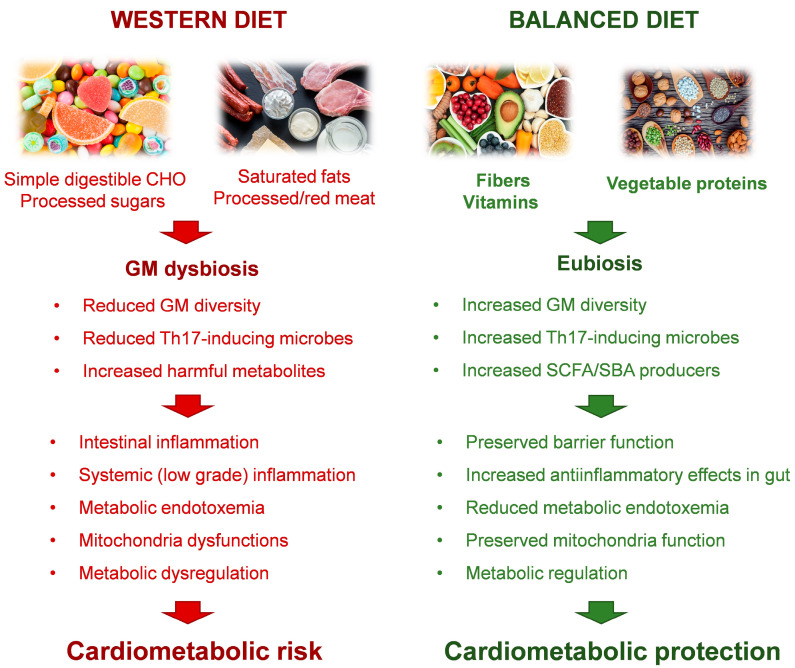
Schematic representation of the impact of different diet components in establishing cardiometabolic status via interaction with gut microbiota.

Dietary substrates directly affect the relative and absolute abundance of gut bacteria as well as their growth kinetics (Figure 1).

### 2.1. Carbohydrates

Dietary carbohydrates (CHOs) are classified as digestible and nondigestible. The former can be degraded by human digestive enzymes to provide energy. Nondigestible CHOs can be divided into fermentable and nonfermentable fibers. Fermentable fibers, glycans, such as pectins, β-glucans, β-fructans, inulins, oligosaccharides, and some resistant starches are fermented by the intestinal microbiota, producing a variety of beneficial substances [12].

Among the macronutrients, the cardiometabolic effects of dietary CHOs are best characterized (Figure 1). In experimental animal models, simple digestible CHOs, such as sucrose or fructose, both alone and as part of a Western-style high-fat high-sugar diet, cause rapid GM remodeling, inflammation, metabolic dysfunction and arrhythmogenesis [13,14,15,16]. High sugar intake promotes an overgrowth of Proteobacteria in the gut, while simultaneously decreasing the abundance of Bacteroidetes. Given that the latter can mitigate the effects of endotoxin, as well as reinforce epithelial integrity and gut barrier function, high sugar-dependent gut dysbiosis promote metabolic endotoxemia, systemic (low grade) inflammation and the development of metabolic dysregulation. As recently demonstrated, the underlying processes deal with a sugar-driven depletion of Th17-inducing microbes promoted by an outgrowth of *Faecalibaculum rodentium* [15]. High-fructose diets cause heart–gut axis disorders that promote cardiac arrhythmia through potentiating proinflammatory mediator, such as tumor necrosis factor alpha (TNF-α), nuclear factor-κB (NF-κB), and interleukin 6 (IL-6) [16].

Complex CHOs, mostly derived from plant but also animal, fungal and algal sources, present a diverse array of monosaccharide linkages, many of which are indigestible by humans. Indeed, the human genome encodes a limited number of carbohydrate-active enzymes (CAZymes) [17]. Thus, glycans, such as resistant starch, inulin, lignin, pectin, cellulose and fructo-oligosaccharides (FOS) reach the large intestine in their undigested forms. On the other hand, GM possesses several other CHO-degrading enzymes and thus use indigestible CHOs as its primary energy source [18]. Bacteria that can degrade glycans are termed primary degraders, and include members of the Bacteroides, Bifidobacterium and Ruminococcus genera [18]. The cardioprotective factors short-chain fatty acids (SCFAs) are the primary end products of bacterial fermentation of glycans, and represent an excellent example of mutualism between humans and their bacterial symbionts (See Section 2.2). Microbiota-accessible CHOs provide a critical energy source for GM, and the consequent production of SCFAs benefits the host by serving as both recovered energy from otherwise inaccessible carbohydrates as well as potent regulatory molecules with vast physiological effects. For example, *Faecalibacterium prausnitzii*, *Roseburia intestinalis*, and *Eubacterium hallii* metabolize dietary fibers as major SCFA producers providing energy sources for enterocytes and achieving anti-inflammatory effects in the gut [19]. In the same way, *Akkermansia muciniphila* exerts beneficial action in metabolic diseases and fortifies the barrier function. Interestingly, fermentable fibers, such as β-glucans, found in oats, and pectin, and in apples, possess bile acid (BA) sequestering activity. This can cause the BAs to travel unabsorbed into the colon where they are excreted or transformed again into secondary bile acids (SBAs) by the colonic bacteria, which results in a net loss of cholesterol [19].

### 2.2. Proteins

In humans, protein breakdown followed by amino acid (AA) absorption in the small intestine is a rather efficient process, however, substantial amounts of amino acids escape assimilation and become available for fermentation by the GM [20]. AAs play a vital role in regulating the diversity and abundance of AA-fermenting microbiota [20] (Figure 1). The most abundant AA-fermenting bacteria in the human small intestine belong to the Clostridium clusters, the Bacillus-Lactobacillus-Streptococcus groups, and Proteobacteria [21]. Lysine or proline are preferred AA substrates of the Clostridium genus while the Peptostreptococcaceae genus are mainly involved in glutamate or tryptophan utilization [22]. Anaerobes including Bacteroides, Lactobacillus, Bifidobacterium, Clostridium, and Peptostreptococcus ferment aromatic amino acids [23]. It is noteworthy that several bacteria species of the genera Fusobacterium, Bacteroides, and Veillonella and the species Megasphaera elsdenii and Selenomonas ruminantium, play a prominent function in AA metabolism in the large intestine [22].

As for CHOs, the influence of proteins on the GM composition is determined by protein type and quantity. Proteins are the primary substrate for both beneficial SCFAs and harmful putrefactive metabolites including ammonia, amines, excessive hydrogen sulfides, phenols, and indoles, that are produced by the GM through proteolytic fermentation and may alter host physiology by influencing the risk of disease [24,25,26,27]. This is best exemplified by the production of the detrimental molecule Trimethylamine-*N*-oxide (TMAO) from the amino acid L-carnitine, which is abundant in animal but not in vegetable proteins. A series of studies in murine models demonstrates that meat and plant foods differentially affect the growth of microbial populations [28,29]. For example, pathogenic classes of the Firmicutes phylum, such as Clostridia and Bacilli, are increased in rats fed with proteins from beef, pork, or fish. On the contrary, soy proteins are considered a rich source of all essential AAs that preferentially support the growth of Lactobacilli and Bifidobacteria and decrease pathogenic taxa involved in metabolic disease [28,29,30,31]. Accordingly, plant protein derived for example from rice, wheat, and, above all, soy have gained wide popularity due to their health-promoting effects against CMD, obesity and diabetes [30,31]. The impact of dietary protein on gut microbial composition has also been comprehensively investigated in several human studies in which participants received animal-based protein from meat, eggs, and cheeses, such as whey protein, or purely vegetarian sources, such as pea protein [32,33,34]. Consumption of plant proteins has been reported to increase gut commensal Bifidobacterium and Lactobacillus in association with increased SCFAs levels, and to decrease the pathogenic *Bacteroides fragilis* and *Clostridium perfringens* [35,36]. On the contrary, bile-tolerant anaerobes, such as Bacteroides, Alistipes, and Bilophila were increased with consumption of animal-based protein [32,34]. This observation is further supported by an independent work comparing the microbiota of Italian children, who ate more animal protein, with that of children in a rural African village [37]. While at recent times, there is a strong interest in advocating plant proteins as a healthier dietary option [38], several studies highlight the importance of animal proteins in the human diet. Animal proteins are considered to be of higher quality since they possess a more balanced essential AA content than vegetable proteins [39,40]. Furthermore, the proteins from animal-based food sources may have beneficial effects on the GM due to the higher digestibility, while the digestion of plant proteins may be limited by the presence of antinutritional factors found in plants [39,41]. It has been demonstrated that moderate dairy and meat protein intake, as recommended by the Mediterranean diet, increases the abundance of the genus Lactobacillus and maintains a more balanced composition of GM compared to soy proteins, with beneficial effects on the host [28,42]. Collectively, these data highlight that the diverse effects of protein diet sources on microbiota-composition and function represent an area ripe for future research.

### 2.3. Fat

The quantity and quality of dietary fat influence the composition of the intestinal microbiota [43]. A GM modified by a diet rich in saturated fat is characterized by the over-representation of lipopolisaccharide (LPS)-expressing bacteria, leading to intestinal inflammation and endotoxemia both in mice and humans [44,45,46] (Figure 1). A high-saturated fatty acid diet (HFD) can also stimulate the production of sulfate-reducing bacteria, leading to defects in the mucous layer and aggravating intestinal inflammation [47]. Gut dysbiosis associated with a HFD exacerbates CVD. For example, western HFD can irreversibly reduce the microbiota diversity, affecting key microbial oscillators and disrupting the host’s circadian rhythm and metabolism, thereby promoting obesity [48]. In addition, the Western HFD is rich in choline that the GM can transform to induce an increase in TMAO levels, thereby promoting the development of CVDs [49]. Concordantly, mice fed an isocaloric diet rich in long-chain saturated fats, derived primarily from meat products, displayed greater insulin resistance and adipose tissue inflammation as compared with that of mice fed a high–fish oil diet [50]. These metabolic alterations were accompanied by reductions in phylogenetic diversity in mice fed with saturated fats, which were reverted by the transplantation of microbiota from mice fed fish oil [50]. Furthermore, transgenic mice that constitutively produce ω3 polyunsaturated fatty acids (PUFA) possess a microbiome with enhanced phylogenetic diversity that offers protection against the metabolic consequences of a high-saturated-fat, high-sugar diet [51]. A comparison of mice on a variety of diets (low-fat diet and diets containing high levels of saturated fat, *n*-*6* PUFA or *n*-*3* PUFA) showed that saturated fatty acids (SFAs) or *n*-*6* PUFA induced weight gain, but only SFAs increased insulin resistance, colonic permeability, and mesenteric fat inflammation [52]. These data are in line with the latest version of dietary guidelines provided by the US Departments of Agriculture and Health that no longer call for a reduction in total fat intake but rather for optimization of fat types in the diet, and specifically reduced intake of saturated and trans fats [53]. Some possible mechanisms by which SFAs could affect GM composition have been suggested. For example, SFAs have a wide spectrum of antibacterial activity including lysis and solubilization of GM membranes [54,55]. Interestingly, the disruptive effect of fat on beneficial microbiota species crosses generations, as the offspring of HFD-fed primates or rats also harbor a dysbiotic gut microbiome [56,57,58], which is probably related to induction of persistent epigenetic modification in genes relevant for tissue development and metabolism [58]. In addition, a HFD impairs mitochondrial uptake of oxygen into host enterocytes and elevates nitrate in the mucus, which in turn weakens healthy anaerobic gut function. Facultative anaerobes, such as the pathobiont *Escherichia coli*, become dominant, which leads to an overall increase in the amount of choline catabolized into the precursor for TMAO [48].

### 2.4. Vitamins

Like macronutrients, even micronutrients, such as vitamins, needed by the body in small quantities, can elicit changes in GM structure and function. The levels and type of vitamins promote the prevalence of some types of microbes at the expense of others through direct and indirect mechanisms collectively increasing or maintaining microbial diversity, affecting the production of GM metabolites, and modulating the gut immune response or barrier function [59] (Figure 1). Direct mechanisms are generally driven by water-soluble vitamins that serve as coenzymes in metabolic reactions necessary for the energy production of bacteria with effects on microbiome growth and composition. For example, in a pilot study by Pham and Coll, vitamin C was found to be the most effective in modulating the human GM in terms of metabolic activity and bacterial composition. In particular, vitamin C significantly increased microbial alpha diversity (evenness) and fecal SCFAs levels compared to the placebo [60,61]. Few works have assessed the role of vitamin B family on GM composition. A pilot study analyzed the effects of vitamin B2 supplementation on the fecal microbiome in 11 healthy adults [62]. Investigators found an increase in the number of *Faecalibacterium prausnitzii* per gram of feces during 2 weeks of supplementation, and a decrease after supplementation, although concentrations did not return to baseline. *Faecalibacterium prausnitzii* has recently attracted interest as the major butyrate producer in the human microbiome, and because of its anti-inflammatory and gut barrier function-improving properties; therefore, vitamin B2 supplementation may directly influence GM composition to provide host benefits [62].

Vitamins can also impact the GM indirectly by modifying the host immune response, the barrier function or the susceptibility to infection [63]. For instance, vitamin A increases microbial diversity of the human GM and the maladaptive changes in microbe structure associated with vitamin A deficiency adversely impact the gut immune response or barrier function, thus, indirectly influencing gastrointestinal health [64]. The retinoic acid, (RA), the active form of vitamin A, is fundamental for the development of the immune system [65], as it stimulates the migration of immune cells including dendritic cells, T cells, and B cells to the intestine and helps execute their function [66]. RA regulates the levels of antimicrobials as well as secretory immunoglobulin A, which in turn influences the gut microbial diversity [67]. Vitamin A deficiency results in increased susceptibility to enteric infections which may be the result of a reduced colonization resistance as a consequence of an altered GM [68]. It was demonstrated that vitamin A-deficient mice are more vulnerable to Citrobacter, a murine pathogen with pathogenetic mechanisms closely related to the clinically important human *Escherichia coli* [69].

In a systematic review on clinical studies, vitamin D supplementation was found to be associated with a significant change in microbiome composition, with main changes in the Firmicutes, Actinobacteria and Bacteroidetes phyla [70]. In addition, a high nutritional intake of vitamin D seems to induce a shift in bacterial composition and affects the species’ richness with a reduction in Veillonellaceae and Oscillospiraceae families, in the Firmicutes phylum with increasing levels of vitamin supplementation. In line with this, emerging evidence suggests that vitamin D deficiency links to CMD through GM [71] and that vitamin D status may play a role in regulating the GM composition by inhibiting the growth of pathogenic bacteria while nourishing the beneficial strains [72].

Collectively, vitamins have a pivotal role in shaping the structure and function of gut microbial community which should be taken into account in the formulation of dietary plans.

## 3. GM-Derived Metabolites

**Figure 2:**
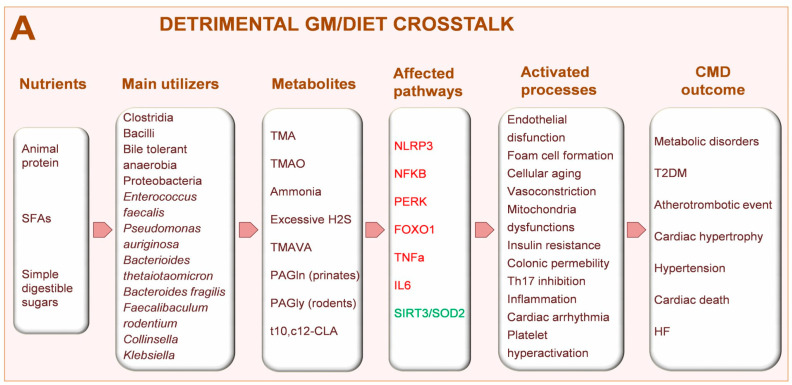

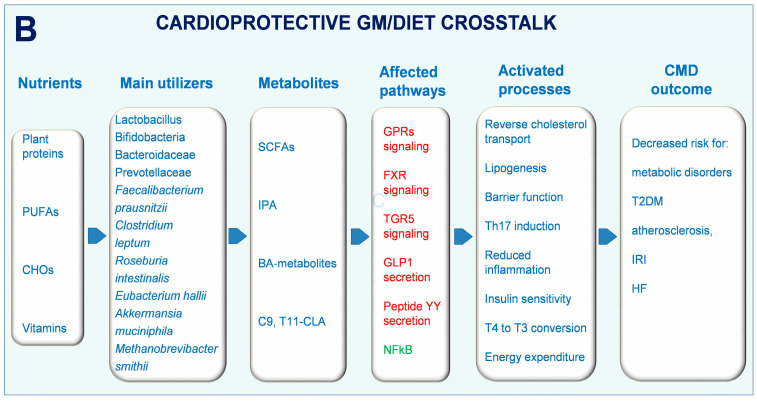
Detrimental (**A**) or protective (**B**) consequences of the GM/diet crosstalk on CMD outcome. Activated pathways are indicated in red font while green font refers to repressed pathways. BA: bile acids; CHOs: carbohydrates (non-digestible); CLA: conjugated linoleic acid; FXR: farnesoid nuclear receptor; FOXO1: forkhead box protein O1; GLP1: glucagon-like peptide 1; GPRs: free fatty acid receptors; HF: heart failure; H2S: sulfhydric acid, IL6: interleukin 6; IPA: indolepropionic acid; IRI: ischemia reperfusion injury; NFkB: nuclear factor kappa B subunit 1; NLRP3: pyrin domain-containing-3; PAGln: phenylacetylglutamine; PAGly: phenylacetylglycine; PERK: protein kinase R-like endoplasmic reticulum kinase; SCFAs: short-chain fatty acids; SIRT3: sirtuin 3; SOD2: superoxide dismutase 2; T2DM: type 2 diabetes mellitus; Th17: t helper 17 cells; TGR5: bile acid receptor 1; TMA: trimethylamine; TMAO: trimethylamine-*N*-oxide; TMAVA: N,N,N-trimethyl-5-aminovaleric acid TNFα.

The enzymatic activities of the gut microorganisms on nutrients introduced with the diet produce a wide variety of bioactive metabolites that affect host physiology by acting locally in the gut or reaching distant organs via the systemic circulation. The rapidly expanding list of compounds that are derived from bacterial metabolism includes many methylamines, polyamines, polysaccharides, SCFAs, SBAs, B vitamins, uremic toxins like *p*-cresol sulfate and indoxyl-sulfate, 4-ethylphenylsulfate, dihydrodigoxin, and a long list of xenobiotic-derived metabolites [4,73,74,75]. Recent advances in the fields of microbiome and metabolome analyses in well-characterized clinical cohorts, have discovered several human CMD-associated gut microbial metabolites [76,77]. Currently, the major classes of GM-dependent metabolites linked to CVD risk, either in animal models or in humans, are trimethylamines, SCFA, SBAs, AA metabolites, and linoleic acid derivatives (Figure 2A).

### 3.1. Trimethylamine-N-oxide

The production of TMAO is an example of GM and host metabolism intertwining. In this process, host and microbe cooperate, in what is termed meta-organismal metabolism, to generate bioactive molecules with significant clinical relevance. TMAO is produced by the liver microsome flavin-containing monooxygenases 3 (FMO3), starting from the GM-derived trimethylamine (TMA) [78]. Several distinct GM enzyme complexes contribute to TMA synthesis, including the choline utilization TMA lyase system (CutC/D), the carnitine Rieske-type oxgenase/reductase system (CntA/B), or the YeaW/X system which can utilize multiple substrate sources [11]. Then, TMA enters the portal circulation and is further processed by the host liver to produce TMAO. Several clinical and experimental studies confirmed that high-TMAO producers include Clostridiales of the Firmicutes phylum, with Clostridiaceae, Lachnospiraceae, and Veillonellaceae being the most abundant (Figure 2A). On the other hand, low-TMAO producers are represented by Bacteroidales within the Bacteroidetes phylum, of which Bacteroidaceae and Prevotellaceae are the most prevalent [79,80,81] (Figure 2B). Since its discovery and first reported link to CVD pathogenesis [11], TMAO has quickly gained attention as both a promoter of atherothrombotic events and biomarker for human CVD [4,82,83,84,85,86,87,88,89,90,91]. Concordantly, dietary provision of TMAO can promote atherosclerosis and thrombotic vascular disease in mouse models [4,82,83,84,85,86,87,88,89,90,91]. TMAO-dependent noxious effects have also been implicated in other close-related CMD including cardiac hypertrophy, cardiac fibrosis, type 2 diabetes, obesity, and heart failure [92,93,94,95,96,97,98,99,100] (Figure 2A). Convincing in vivo and in vitro evidence indicates that TMAO exerts a broad range of actions in diverse processes, such as endothelial dysfunction, foam cell formation, thrombosis, and cholesterol metabolism (see Ref. [101] for a comprehensive rev.). In human umbilical vein endothelial cells, TMAO induces markers of cell dysfunction, aging and monocyte adhesion, which is associated with the activation of pyrin domain-containing-3 (NLRP3) inflammasome, increased production of reactive oxygen species (ROS) and perturbation of the SIRT3-SOD2-mitochondial ROS signaling pathway [102,103,104]. In human coronary artery vascular endothelial cells and in smooth muscle cells, TMAO promotes the proinflammatory mitogen activated protein kinase (MAPK) and NF-kB signaling cascade and foster the production of tissue factor, a protein implicated in the thrombogenicity of atherosclerotic plaques [105,106,107,108]. Also, TMAO favors cholesterol uptake in macrophages by increasing the cell surface expression of proatherogenic receptors, such as cluster of differentiation 36 (CD36) and ATP-binding cassette transporter A1 [9,109]. However, some intervention studies have demonstrated that foods generally associated with significant reduction in CMD risk, such as fish, polyphenols, and whole-grain cereals, also prompt the production of TMAO [110,111,112]. Such counterintuitive findings can be explained, at least in part, by considering that the healthful components of the diet might have counterbalanced or even exceeded the negative effects of TMAO on CVD risk.

Although the available studies have provided valuable information about the molecular events underlying the biological effects of TMAO, the precise nature of the molecular mechanism driving TMAO-mediated CVD are still being elucidated, due to the lack of knowledge on the molecular sensor for TMAO. In this regard, a recent study has revealed that TMAO, at physiologically relevant concentrations, directly and selectively interacts with protein kinase R-like endoplasmic reticulum kinase (PERK) protein, one of the three key molecular sensors in endoplasmic reticulum involved in the unfolded protein response signaling. This interaction selectively activates the PERK branch of the unfolded protein response, and induces the transcription factor Forkhead box protein O1 (FOXO1), a key promoter of metabolic disease, which can be prevented by interventions to reduce TMAO. Taken together, these data suggest that TMAO and PERK may be central to the pathogenesis of the metabolic syndrome [113].

In recent years, experimental findings have suggested that, apart from being the precursors of TMAO, TMA may affect the CV risk by directly promoting vascular contraction and hypertension [114,115] (Figure 2A). A better understanding of the relationship between TMA and the CV system with further preclinical and clinical studies may bring novel insights into the diagnosis and therapy of CMD.

### 3.2. Short-Chain Fatty Acids

In healthy individuals, the GM is enriched for taxa exhibiting an increased capacity for the production of SCFAs (acetate, butyrate, and propionate), which favors intestinal integrity and metabolic homeostasis [7,116]. The main SCFA producing-bacteria in the human gut belong to the phylum Firmicutes, in particular *Faecalibacterium prausnitzii* and *Clostridium leptum* of the family Ruminococcaceae and *Eubacterium recta*, and *Roseburia* of the family Lachnospiraceae [117,118]. In addition, sugar-and/or lactate-utilizing bacteria produce butyrate from lactate and acetate, such as *Eubacterium hallii* and Anaerostipes [117] (Figure 2B). SCFAs play an important cardiometabolic protective role through modulating a range of physiological processes, including energy homeostasis, lipid and carbohydrate metabolism, host immune system composition, and suppression of proinflammatory signaling. Indeed, SCFAs stimulate the secretion of glucagon-like peptide 1 (GLP1) and peptide YY by intestinal L cells to control glucose homeostasis and regulate food intake [119,120], and induce the differentiation of anti-inflammatory and pro-reparative regulatory T cell (Treg) [7,121]. The beneficial effects of SCFAs are produced via G-protein coupled receptors (GPCRs) along with gut–brain neural circuits [122,123], and involve epigenetic regulation of host gene expression [121] (Figure 2B). The main receptors for SCFAs are free fatty acid receptor (FFAR) 2 and 3 (also known as GPR41 and GPR43), and GPR109A, that are expressed in intraepithelial lymphocytes of the gastrointestinal tract, immune cells, colonic epithelial cells, sympathetic ganglia, and adipose tissue lymphocytes [120]. It has been demonstrated that propionate acts as an agonist of FFAR3 in the periportal afferent neural system to induce intestinal gluconeogenesis (IGN) via a gut–brain neural communication [122], while butyrate can directly activate IGN gene expression through a cAMP-dependent mechanism [123]. The glucose released by IGN is detected by a portal vein glucose sensor that transmits its signal to the brain via the peripheral nervous system to promote beneficial effects on food intake and glucose metabolism [124]. Thus, IGN represents a signal to the brain that food previously ingested is enough for maintaining plasma glucose [125]. In addition, sensing of SCFAs by FFAR2, FFAR3, and GPR109A affects lipid and sugar metabolism, while inhibiting inflammatory pathways [123,126]. These processes rely, at least in part, on epigenetic mechanisms in colon macrophages and epithelial cells. SCFAs can be directly converted (acetate) or oxidized (propionic and butyrate) to acetyl-CoA, the substrate of the histone acetyltransferase (HAT) enzymes, thus affecting host gene expression. For example, the main mechanism by which butyrate promotes Treg differentiation is by an histone acetylation-dependent induction of the immune modulator Forkhead bOX P3 (FOXP3) in naïve T cells [127]. In vivo studies have indicated that butyrate exerts anti-inflammatory effects by inhibiting histone deacetylases (HDACs) in colon macrophages which is related to GRP41/43 activation and reduced levels of IL-6 and IL-8 cytokines [126].

### 3.3. Bile Acid-Derived Metabolites

Bile acids are traditionally considered to facilitate assimilation of fats and fat-soluble vitamins, however, they also play a pivotal role in the crosstalk between GM and CV health. Primary bile acids (PBAs), such as cholic and chenodeoxycholic acid (CDCA), are synthesized in the liver from oxidation of cholesterol [19] and conjugated to amino acids taurine or glycine to form bile salts that are secreted into bile and stored in the gallbladder. They are released into the small intestine in response to food intake to emulsify fats and form micelles that are absorbed by the enterocytes [19]. More than 95% of the conjugated and unconjugated PBAs are typically reabsorbed [128]. The remaining are significantly processed in the gut by bacterial enzymes [129]. Specifically, bile salt hydrolase, bile acid inducible (BAI) and bile acid dehydratase enzymes, expressed by certain species of gut bacteria, can modify PBAs to generate unconjugated and SBAs, such as deoxycholate lithocholate (LCA), ursodeoxycholate (UDCA), and others [130]. Some species of SBAs are less reabsorbed and consequently may be excreted in the stool, leading to a net loss of cholesterol. Since high circulating low density lipoprotein-cholesterol (LDL-C) levels are a significant risk factor for CVD, BA metabolism may provide a link between the GM and CV health. BA metabolizing gut bacteria are Clostridium, Enterococcus, Bifidobacterium, Lactobacillus and members of the genus Bacteroides (Figure 2B).

Our understanding of BA physiology has been greatly improved by the discovery of SBA responsive receptors, such as farnesoid nuclear receptor (FXR) [131], vitamin D receptor (VDR) and various GPCRs, including bile acid receptor 1 (TGR5), and sphingosine phosphate receptor [132,133,134] (Figure 2B). Among these, FXR is the most widely investigated BA receptor signaling that modulates metabolism and inflammation [135]. FXR seems to decrease triglyceride levels via reducing lipogenesis and promoting increased uptake, catabolism and oxidation of triglycerides and fatty acids [136]. Also, activation of FXR has been shown to reduce plasma glucose levels and improve insulin sensitivity in several models of obesity and diabetes [135]. In atherosclerotic disease, FXR signaling has been shown to antagonize inflammatory responses through repression of NF-kB [137]. In addition, treatment with the FXR agonist CDCA decreased blood pressure in spontaneously hypertensive rats (SHRs) by improving vasorelaxation and reducing vasoconstriction [138]. Meanwhile, the SBAs, LCA and DCA act as powerful ligands for TGR5 to influence several important metabolic pathways, such as thermogenesis, energy metabolism and glucose homeostasis [19]. Activation of TGR5 leads to increased intestinal production of the gut hormone GLP1, promoting insulin secretion and regulation of appetite, and increased energy expenditure via conversion of the thyroid hormone T4 into the active form, T3 [139,140] (Figure 2B).

### 3.4. Amino Acids and Their Metabolites

In addition to the utilization of AAs, the GM performs a key function in AA production, including essential AAs [141], which can impact host physiology. For instance, several AAs generated in the large intestine from microbial protein fermentation provide precursors for the synthesis of SCFAs [142].

Besides being used for the production of bacterial components, AAs are catabolized by GM through different pathways resulting in the formation of bioactive compounds with beneficial or adverse impact on the host. A recent paper demonstrated that *Clostridium sporogenes*, from the phylum Firmicutes, uses aromatic AAs substrates to generate several compounds, one of which, indolepropionic acid (IPA), plays a key role in fortifying the intestinal barrier through the inhibition of the toll like receptor 4 (TLR4) signaling [143,144] (Figure 2B). Lactobacillus can also use tryptophan to produce indole metabolites offering mucosal protection from inflammation [145]. In animal models, microbially-produced IPA is involved in reducing atherosclerosis risk via promoting reverse cholesterol transport, while in patients with coronary artery disease (CAD), IPA levels are significantly associated with decreased risks of cardiovascular and all-cause mortality [146,147].

On the contrary, N,N,N-trimethyl-5-aminovaleric acid (TMAVA), derived from trimethyllysine through the intestinal bacteria *Enterococcus faecalis* and *Pseudomonas aeruginosa* [148], has adverse effects on the cardiovascular system [140] (Figure 2A). In a prospective heart failure (HF) cohort, higher plasma TMAVA levels were associated with greater incidents of cardiac death and transplantation risk, independent of traditional risk factors. In mice on a HFD, TMAVA aggravated cardiac hypertrophy and dysfunction via mitochondrial structural and functional impairment, reduced oxidation of free fatty acids, and increased myocardial lipid accumulation and toxicity [149]. Phenylacetylglutamine (PAGln) is another GM-derived metabolite associated with CVD (Figure 2A). The first step in the generation of PAGln is the gut bacteria-dependent transformation of phenylalanine into phenylpyruvic acid (PPY) and subsequently into phenylacetic acid (PAA) [150]. Following absorption into the portal circulation, host hepatic and renal enzymes catalyze conjugation of PAA to either glutamine forming PAGln (major pathway in primates) or glycine forming phenylacetylglycine (PAGly, major pathway in rodents). PAGln is associated with atherothrombotic heart disease, HF and major adverse cardiovascular events in humans [150,151]. It is also causally linked to cardiovascular disease pathogenesis in animal models via the activation of α2A, α2B, and β2-adrenergic receptors on platelet surface leading to platelet hyperactivation [150]. Moreover, mechanistic studies showed that both PAGln and its murine counterpart, phenylacetylglycine, directly fostered HF-relevant phenotypes, including decreased cardiomyocyte sarcomere contraction, and B-type natriuretic peptide gene expression in both cultured cardiomyoblasts and murine atrial tissue [151]. A recent paper describes two different gut microbial pathways for the formation of PAA, one catalyzed by the *Bacteroides thetaiotaomicron* phenylpyruvate/ferredoxin oxidoreductase, and the other by the *Proteus mirabilis* phenylpyruvate decarboxylase [152]. Metagenomic analyses reveal a significantly higher abundance of both pathways in gut microbiomes of atherosclerotic CVD patients compared with controls [152]. Given the numerous links between PAGln and CVD, these findings might assist future efforts to therapeutically target PAGln formation in vivo.

### 3.5. Conjugated Linoleic Acids

GM can process double bonds of the unsaturated linoleic acid (LA) to produce metabolites that cannot be synthesized by mammalian hosts, but have the capacity to influence host physiology and health [153]. Several gut bacteria including Lactobacillus, Butyrivibrio, and Megasphaera can produce different conjugated linoleic acid (CLA) isomers with opposite effects on cardiometabolic risk factors [54] (Figure 2). For example, t10,c12-CLA worsens atherosclerosis and insulin sensitivity by inhibiting the expression of peroxisome proliferator-activated receptor gamma (PPARγ) and liver X receptor alpha (LXRα) [154]. On the contrary, c9,t11-CLA and t9,t11-CLA reduce atherosclerosis and improve insulin sensitivity by enhancing PPARγ and LXRα [54]. Given that different bacteria produce different ratios of CLA isomers [155,156], favoring high levels of beneficial CLA-producers could potentially be used to promote a healthy metabolic phenotype.

Bacterial production of CLAs is a multistep process involving several metabolic intermediates. Among these compounds, several hydroxy fatty acids can affect the processes related to host health. 10-oxo-cis-12-octadecenoic acid (KetoA) increases adiponectin production and glucose uptake in a PPARγ-dependent manner, and contributes to the prevention of obesity-related metabolic perturbations [157]. Another hydroxylated CLA intermediate, 10-hydroxy-cis-12-octadecenoic acid (HYA), enhances intestinal barrier function by restoring tight junction activities in a free fatty acid specific receptor GPR40-dependent manner [158]. A successive work in HFD-fed mice determined that HYA is not only the initial PUFA metabolite derived from LA, but also the most important gut microbial PUFA-metabolite that influences host metabolism and confers resistance to obesity [159]. It is noteworthy that conversion of excessive dietary LA to HYA by the GM suppresses inflammatory responses and exerts beneficial metabolic effects via regulating intestinal environment rather than through a systemic action [159].

## 4. GUT Dysbiosis and CVD

In addition to providing substances harmful for the host, gut dysbiosis, favored by an unhealthy diet, can impact the physiology of the cardiovascular system by directly engaging the host innate immune system and thus promote inflammatory pathways [11]. The bacterial host cross-talk is directed by the interaction of microorganism associated molecular patterns (MAMPs) with the host pattern recognition receptors (PRRs). MAMPs include surface antigens present on the bacterial wall as well as a variety of small molecules produced by the GM. Increasing evidence points at the involvement of MAMPs/PRRs signaling in the development of CMD [160]. For instance, the LPS found on the outer membrane of Gram-negative bacteria is a classic MAMP that interact with TLR4 in multiple tissues and cell types, such as macrophages, dendritic cells, cardiomyocytes and cardiac fibroblasts, to prompt the inflammatory response and adverse remodeling of the myocardium [160]. The proinflammatory nucleotide-binding oligomerization domain 1 (NOD1) protein, that sense conserved motifs in bacterial peptidoglycans [161], has been involved in atherogenesis and endothelial inflammation in mice model, human coronary endothelial cells, and patients [162,163,164]. Other main PRR-signaling pathways able to influence atherogenesis and CVD risk factors in clinical arena and murine animal models are the double stranded RNA receptor, TLR3 [165,166]; the bacterial diacylated and triacylated lipopeptide sensors, TLR2/TLR6 heterodimeric complex [167,168]; the unmethylated CpG DNA receptor, TLR9 [168,169]; and the bacterial flagellin sensor, TLR5 [170]. On the basis of specific cytosolic adaptor proteins recruited to TLRs, TLR signaling can be divided into two general cascades. The first one relies on the myeloid differentiation factor 88 (Myd88) and is typical of almost all the TLRs [171]. The second route, termed Myd88 independent, is specific for TLR3 and is mediated by the TIR domain-containing adaptor-inducing interferon-*β* (TRIF), and the TRIF-related adaptor molecule (TRAM) [171]. These signals culminate in the activation of numerous transcription factors, including the nuclear factor-κB (NF-κB) and the interferon regulatory factors (IRFs), to elicit the expression of proinflammatory cytokines and interferons (IFNs), respectively [171]. In turn, maladaptive inflammation accelerates the onset and progression of CMD.

### 4.1. Metabolic Syndrome

Several metagenomic shotgun-sequencing studies have characterized the changes to GM taxa and function in type 2 diabetic and obese subjects [6,114,172,173,174,175]. The main alterations include a depletion of SCFA producing bacteria and an increased potential for the production of excessive hydrogen sulfide and LPS, all associated with metabolic dysfunction and inflammation [113,172,174] (Figure 2A). Interestingly, in individuals with type 2 diabetes treatment with the anti-diabetic drug metformin results in a decreased abundance of *Intestinibacter* spp. and an increased abundances of species of the Enterobacteriaceae family, such as *Escherichia coli*, compared with untreated individuals, which correlates with an increased secretion of GLP1 [173].

A lower gut microbial diversity, in addition to a higher Firmicutes-to-Bacteroidetes ratio, has also been described in obese individuals [113]. Metagenomic sequencing of fecal samples in a cohort from Denmark, which was stratified into obese (body mass index (BMI) > 30), overweight (BMI 25–30) or lean (BMI < 25) individuals, shows that the GM of obese individuals is more prone to present low gene counts [174]. The differences in gene richness seem to correspond to a depletion of butyrate-producing bacteria, such as *Faecalibacterium prausnitzii*, *Butyrivibrio* spp. and *Roseburia inulinivorans*, as well as *Akkermansia* spp. and the methanogenic archaeon *Methanobrevibacter smithii*, whereas *Bacteroides* spp. and *Ruminococcus gnavus* were more abundant in these gut microbiomes with lower gene richness. Furthermore, this study shows that the species composition of the GM based on nine bacterial strains may be used as a disease classifier that better distinguish between lean and obese individuals than host genetic factors. According to this report, analyzing alterations in the microbial gut metagenome may define subsets of adult individuals with different metabolic risk profiles and thereby contribute to resolving some of the heterogeneity associated with adiposity-related phenotypes [116]. A more recent metagenomic analysis in 1545 subjects from the MetaCardis cohorts indicates that severe obesity is associated with an absolute deficiency in bacterial biotin producers and transporters, whose abundance is associated with host metabolic and inflammatory phenotypes. This finding correlates with suboptimal circulating biotin levels and altered expression of biotin-associated genes in human adipose tissue [176]. Mechanistic experiments in HFD-fed murine models including germ-free and antibiotic treated animals, fecal microbiota transfer, bariatric surgery and supplementation with biotin, demonstrate the causal contribution of GM to host biotin levels and weight gain [176]. Therefore, strategies combining biotin and prebiotic supplementation could help prevent the deterioration of metabolic states in severe obesity.

### 4.2. Atherosclerotic Cardiovascular Disease

Gut dysbiosis may influence the circulatory system and atherosclerotic cardiovascular disease (ACVD) evolution through indirect or direct signalings. First, gut bacteria and/or their metabolites may stimulate the enteric nervous system, which in turn modulates the activity of the brain centers controlling the cardiovascular physiology [177]. Secondly, GM-dependent endotoxemia can affect the function of vessel endothelial cell, vessel wall, and blood cells favoring ACVD. For example, inducing gut dysbiosis by *Bacteroides fragilis* supplementation in HFD-fed mice prompts a reduction in Lactobacillaceae and leads to the deterioration of glucose/lipid metabolic and inflammatory responses, which likely promotes aorta plaque formation and the progression of atherosclerosis [178] (Figure 2A).

Pioneering studies in individuals with established atherosclerosis have analyzed microbiota communities in oral, gut and atherosclerotic plaques reporting a reproducible correlation between disease and bacterial pathogens including *Chlamydia pneumoniae*, *Porphyromonas gingivalis*, *Helicobacter pylori* and *Aggregibacter actinomycetemcomitans* [179,180,181]. Importantly, besides the intestinal tract, some of these species have been also found within atherosclerotic plaques, which is consistent with a possible engagement of PRRs in several distinct microenvironments [11,180,181]. A gut metagenome-wide association study (MWAS) identifies the genus *Collinsella* spp. as an enriched species in subjects with atherosclerosis, while *Eubacterium* spp. and *Roseburia* spp. are more abundant among healthy controls [182]. A more recent MWAS on stools from 218 individuals with ACVD and 187 healthy controls confirmed a deviation of the ACVD gut microbiome from the healthy status with an increased abundance of Enterobacteriaceae (including *Escherichia coli*, *Klebsiella* spp., and *Enterobacter aerogenes)*, of bacteria that are often present in the oral cavity (*Streptococcus* spp., *Lactobacillus salivarius*, *Solobacterium moorei*, and *Atopobium parvulum)*, and of *Ruminococcus gnavus*, a bacterium previously associated with inflammatory bowel diseases and low gut microbial richness [76]. In contrast, butyrate-producing bacteria (including *Roseburia intestinalis* and *Faecalibacterium* cf. *prausnitzii*), and common members of the gut microbiome, such as *Bacteroides* spp., *Prevotella copri*, and *Alistipes shahii*, were also relatively depleted in ACVD [76]. From a functional point of view, ACVD gut metagenome is characterized by altered potential for metabolism/disposal of several molecules important for CV health, such as TMA and SCFA production, along with taurine transport [76].

The close correlation between gut dysbiosis and serum metabolite alteration in the setting of coronary artery disease (CAD) has been well documented [77,150]. While the majority of papers are focused on finding new potentially noxious metabolites that are increased in CAD patients, a new multiomic approach in patients with acute coronary syndrome (ACS) found a unique metabolome and GM signature, characterized by significant perturbation in hundreds of metabolites and the lack of multiple serum metabolites, many of which are associated with diet and microbiome [77]. These findings are related to a depletion of the previously unknown bacterial species SGB 4712 of the Clostridiaceae family. By further linking this bacterium with the levels of both cardiotoxic and cardioprotective metabolites, this study demonstrated how the absence of a specific bacterial genome may correspond to an increased risk for CAD, and suggests a concrete target to be evaluated in follow-up intervention studies. Moreover, metabolic deviations in ACS patients linked to microbiome and diet were person specific, and were also observed to a lesser extent in control individuals with metabolic impairment, suggesting the involvement of these aberrations in earlier dysmetabolic phases preceding clinically overt CAD [77].

### 4.3. Acute Myocardial Infarction

The relation between gut dysbiosis and the extent of post myocardial infarction recovery has been explored both in experimental models and in patients [7,183,184,185] (Figure 2A). A mice model of antibiotic-induced gut dysbiosis reports convincing evidence that reorganization of the GM (such as a reduction in Lactobacillus) plays an essential role in adverse post MI cardiac repair and that this may be through the reduction in specific SCFAs responsible for the modulation of the immune system and the inflammatory cardiac microenvironment [7]. Indeed, supplementing antibiotic-treated mice with a Lactobacillus probiotic before MI restores myeloid cell proportions, yields cardioprotective effects, and shifts the balance of SCFAs toward propionate. A successive work on the same model, evidences alteration of indirect producers of butyric acid Prevotellaceae, Clostridiaceae, and Lachnospiraceae after MI, which is related to the induction of HDAC-dependent adverse repair [183]. These studies suggest that manipulating the production/levels of GM-derived SCFAs may provide opportunities to modulate pathological outcome after MI. The results on MI have also been confirmed in acute myocardial infarction (AMI) murine models. For instance, diet-dependent dysbiotic GM in rats contributes to increased myocardial infarct size in correlation with a higher Firmicutes/Bacteroidetes ratio, NF-kB activation, and increased plasma concentration of LPS, which can be attenuated by probiotic administration or non dysbiotic diet [186].

In line with animal studies, association between diet and GM dysbiosis has also been identified in AMI cases. In patients with stable angina, high dietary intake of TMA precursors, choline, phosphatidylcholine and sphingomyelin, are associated with increased risk of AMI [185]. The main GM alterations in AMI cases are lower abundance of SCFA producers including *Tyzzerella 3*, *Dialister*, *Eubacterium ventriosum group*, *Pseudobutyrivibrio*, and *Lachnospiraceae ND3007 group*, reduced abundance of the gut barrier protector *Roseburia homini*, and enrichment of opportunistic pathogenic bacteria including *Parabacteroides merdae*, *Ruminococcus bromii*, and *Ruminococcus torques*, that are associated with increased systemic inflammation [184]. In addition, using these species abundance differences, a gut microbial-based risk score created at disease onset, is associated with the disease prognosis [184]. Among the discriminatory species, *Akkermansia muciniphila*, *E. hallii*, and *Ro Ruminococcus hominis*, were identified to be the most critical classifier for AMI status [184]. Remarkably, differences in fecal GM composition discriminate different subgroups of AMI including ST elevation myocardial infarction (STEMI) vs non STEMI cases [187]. From a functional point of view, the main alterations of GM in the AMI patients are related to the biosynthesis of methionine. Such pathways are associated with systemic inflammation and AMI status and collectively acts as one hub signaling connecting GM and metabolites with cardiometabolic phenotypes [184].

### 4.4. Heart Failure

Several sequencing-based clinical studies report significantly different GM composition and function between HF patients and healthy subjects [188,189,190,191]. A common finding is the relative reduction in taxa from Lachnospironacea (such as Eubacterium), and Ruminococcacea (such as Faecalibacterium) families, known for their ability to produce butyrate (Figure 2). This finding is also supported by the lower enrichment of GM genes relevant to butyrate production [192]. Of note, the abundance of several members of the Lachnospiracea family correlate with soluble CD25, a marker of T cell activation. In addition, depletion of the butyrate producer *Eubacterium Halli* and increased plasma levels of soluble CD25 were associated with death or heart transplantation [191]. Also, increased abundance of several pathogenic bacteria, including Campylobacter, Shigella, Salmonella, *Yersinia Enterolytica* and Candida species are found in association with HF [190]. Changes in the composition of GM in HF have also been confirmed in experimental animal studies, which helped elucidate the role of microbiota in the development of HF [8,192,193].

Different metabolites produced by dysbiotic gut microbes from dietary metabolism have been linked to the pathologies of HF including TMAO, BAs and AAs (Figure 2A). In addition to the strong association between TMAO and major adverse cardiac events [194], this metabolite has recently been implicated in HF through both human and animal model investigations [92,93]. Animal studies have further demonstrated that targeting of the TMAO pathway through either diet or microbial enzyme inhibitors impacts ventricular remodeling in mice [93]. SBAs are another class of microbiota-derived metabolites with evidence for a potential role in HF. Both the composition and pool size of BAs are altered in subjects with HF. For example, a recent small cohort study on HF patients and sex-matched controls, reported a decreased ratio of primary to secondary BAs in diseased subjects [195]. Such a difference was driven mainly by decreased levels of PBAs. Moreover, there was a shift in the SBA profile, though the total SBA level remained similar. In addition, in a small prospective, double-blind, randomized placebo-controlled crossover study, clinically stable patients with HF were reported to have modest improvements in blood flow with administration of the SBA ursodeoxycholic acid [196]. Finally, considerable evidence shows an altered composition of microbiota and AAs in HF patients, and supports the relevance of AAs as powerful diagnostic and prognostic biomarkers in disease progression [197]. A main AA disturbance in HF subjects is the decreased levels of circulating essential AAs (EAAs), especially branched-chain AAs and histidine, which correlates with decreased abundance of microbial genes involved in EAA biosynthesis and increased expression of EAA degrading microbial enzymes [198]. Network analysis revealed that the depletion of Eubacterium and Prevotella, harboring genes for BCAA and histidine biosynthesis, contributed mostly to the observed EAAs reduction. Other major AA metabolism alterations are increased production of TMAVA and PAGln, both clinically and mechanistically linked to HF presence, severity, and mortality risk [149,151] (see also par 1.2.4). All these findings indicate that the gut–AA–HF axis may play a key role in HF etiopathogenesis and could represent a potential therapeutic target.

## 5. Diet Intervention to Target the Heart/GM Axis

The last evidence-based dietary guidance from the AHA to improve cardiovascular health includes the following recommendations: “eat plenty and a variety of fruits and vegetables; choose whole grain foods and products; choose healthy sources of protein (mostly plants; regular intake of fish and seafood; low-fat or fat-free dairy products; and if meat or poultry is desired, choose lean cuts and unprocessed forms); use liquid plant oils rather than tropical oils and partially hydrogenated fats; choose minimally processed foods instead of ultra-processed foods; minimize the intake of beverages and foods with added sugars; choose and prepare foods with little or no salt; if you do not drink alcohol, do not start; if you choose to drink alcohol, limit intake” [2]. Given the low content of TMAO precursors and the high abundance of fibers and prebiotics, such a dietary pattern is expected to favorably impact the GM composition, as described below.

### 5.1. Prebiotics, GM Remodeling and CMD

According to an expert consensus document on the definition and scope of prebiotics, a dietary prebiotic is “a substrate selectively utilized by host microorganisms conferring a health benefit” [199]. The most common prebiotics associated with CV benefits are carbohydrate substrates including fructans, β-glucans, fructose polysaccharide, galacto oligosaccharides and inulin oligosaccharides [19]. Such compounds have been shown to affect the GM profile in humans in a dose-dependent manner with positive effects on CMD risk markers including advanced glycation end-products (AGEs), soluble receptor for AGEs (sRAGE), triacylglycerol, and LDL cholesterol [200,201,202,203]. For example, the cholesterol-lowering effects of oats were associated with increased plasma levels of SCFA and enrichment of *Akkermansia muciniphila*, *Roseburia*, *Bifidobacterium*, and *Faecalibacterium prausnitzii* GM species [197,198]. In addition to favoring the growth of SCFA producers, fiber prebiotics, such as arabinoxylan purified from corn bran, have been shown to exert a satietogenic effect in overweight subjects probably due to bacterial taxa that ferment the fiber or utilize breakdown products [204]. Prebiotics can also affect the growth of certain species with strong BA deconjugating activity to favor the generation of unconjugated and SBAs that are less well absorbed in the intestine and are lost in the feces. Replacing the excreted BAs through BA neo-synthesis represents an effective system to regulate circulating cholesterol level in the body. Accordingly, a recent study on mild hypercholesterolemic subjects reported that increasing BA synthesis rather than inhibiting cholesterol synthesis or absorption may be another mechanism responsible for the cholesterol-lowering effect of high molecular weight prebiotics, such as the burley *β*-glucan [205].

Based on the above-reported definition, polyphenols are another class of crucial secondary metabolites produced by the plant kingdom and included in the group of prebiotics. Therefore, nutritional regimens rich in phenolic compounds, such as fruits and vegetables, are considered to have beneficial effects on gut and CV health [19]. Like fibers, polyphenols reach the gut undigested and are fermented by resident bacteria leading to generation of smaller phenolic compounds that can be absorbed by the intestine and produce anti-inflammatory and vasodilatory systemic effects [19]. Complex polyphenols have been found to prompt the proliferation of GM involved in the deconjugation hydrolysis of PBAs [206]. A study on a murine model of atherosclerosis suggested that consumption of resveratrol can limit the effects of TMAO on CVD development by promoting greater uptake of circulating cholesterol used by the liver to produce new BAs [207]. This effect was due to increased levels of genera with strong bile salt hydrolase activity like Lactobacillus and Bifidobacterium, and reduced abundance of TMA producers. In patients with coronary artery disease, resveratrol consumption did not affect TMAO circulating levels but induced a significant remodeling of the GM with a difference in β diversity and predominance of Parasutterella, Ruminococcaceae, several Bacteroides species, and Prevotella. Interestingly, plasma metabolomic analysis revealed significant changes in metabolites after resveratrol consumption, consistent with improved redox homeostasis [208]. Experimental evidences in diabetic or high fat-fed mice demonstrate that dietary polyphenols contained in berry including blueberry, strawberry, and cranberry support the growth of beneficial bacteria, such as Bifidobacterium, Lactobacillus, *and Akkermansia muciniphila*, which help in metabolizing anthocyanins into small metabolites, like phenolic acids. These compounds in turn ameliorate vascular complication, and vascular inflammation associated with the CMD models [209,210,211]. Similarly, administration of olive leaf extract, rich in polyphenol oleuropein, to HFD-fed mice ameliorated GM dysbiosis while reverting endothelial dysfunction and improving plasma lipid profile [212].

Among other phytochemicals with prebiotic activity, Berberin has been shown to play a major cardioprotective role in an atherosclerosis-prone Apoe^−/−^mice by increasing the abundance of Akkermansia, the beneficial bacteria associated with metabolic health [213].

The interactions of different dietary prebiotic combinations on GM composition and CV health has also been investigated. In mice fed an obesogenic diet, administration of cranberry polyphenols and agavins, a highly branched agave-derived neo fructans, shaped GM composition and regulated key mucosal markers involved in the repair of epithelial barrier integrity, thereby attenuating obesity-associated gut dysbiosis and metabolic inflammation and improving glucose homeostasis. The main stimulated bacteria were *Akkermansia muciniphila* and the glycan-degrading *Muribaculum intestinale*, *Faecalibaculum rodentium*, *Bacteroides uniformis*, and *Bacteroides acidifaciens* [211]. Along the same line, in adults with prediabetes supplementation via polyphenol-dense red raspberries and a fructo-oligosaccharide increased Bifidobacterium, and concurrently reduced *R. gnavus*, and this was associated with metabolic improvements [214].

Collectively, the available experimental and clinical findings suggest that dietary prebiotics may favor a cardioprotective GM remodeling. However, if a better adherence to the dietary guidance is expected to reduce the probability of developing CMD, the existence of person specific differences in response to the same diet highlight the need of more personalized nutritional plans.

### 5.2. Personalized Nutrition

The flourishing of microbiota research has unveiled a major inter-individual variability in GM composition even in healthy people [215]. A study on twins revealed major differences in postprandial lipidemic response to identical meals indicating that non genetic host-specific factors, including GM, play a crucial role in determining the different responses to diet [216]. Along the same line, in CMD subjects, with the exception of certain above-described common traits, such as altered abundance of TMAO and SCFA producers, different GM-related pathways may be identified in different patients, which greatly affect disease susceptibility and response to diet interventions. For example, in an obese cohort subjected to calorie restriction, the baseline abundance of *Akkermansia muciniphila*, the mucin-degrading bacterium, was associated with improved metabolic outcomes [217]. In another study, the baseline GM composition outperformed other host intrinsic factors in affecting the diet-induced individual weight loss, with higher abundance of *Blautia wexlerae* and *Bacteroides dorei* being the strongest predictors for weight loss [218]. These findings indicate that GM composition should provide useful insights in guiding personalized nutritional advice. In this direction, Zeevi et al. performed an elegant study on 800 obese or overweight subjects evidencing the limited utility of universal dietary recommendation given that what represented a good food for some individuals might be detrimental for others and vice versa. On the contrary, the integration of GM, blood parameters, dietary habits, anthropometrics, and physical activity through a machine-learning algorithm accurately predicted personalized postprandial glycemic response to meals. Importantly, when this approach was used to adapt diets to factors underlying inter-individual differences, considerable changes were observed in postprandial glucose levels and GM configuration [175]. The same personalized predictive model has been independently validated in non-diabetic patients confirming that combining clinical characteristics, physiological variables with GM and diet is more predictive than current dietary approaches focusing only on the calorie or carbohydrate content of foods [219]. Another clinically applicable method is based on the so-called oral carnitine challenge test. The integration of the pharmacokinetic study with GM, serum biochemistry, host genotypes, and dietary records successfully identified TMAO producer phenotype, which may help personal nutritional guidance in the prevention and treatment of CVD [81]. Personalized nutrition at individual levels requires the collection of a huge amount of data, which is both costly and time consuming. Metabotyping has been suggested as a more feasible procedure for precise prevention of CMD [220]. The idea behind the concept is to group individuals according to metabolic phenotypes based on factors, such as GM, anthropometrics, clinical parameters, metabolomics data, and diet. In such a way, an optimal nutritional regimen can be tailored to specifically target each metabotype.

All of these approaches are at their infancy, and face significant challenges towards large scale implementation in humans. The simplification of sample processing, along with standardization of data collection and analytical procedures may improve accessibility to such data-driven nutritional advice.

## 6. Conclusions and Future Perspectives

Accumulating evidence demonstrate a complex cross-talk linking GM, dietary components, and CM risk and disease. It is well documented that unhealthy nutritional habits favor intestinal dysbiosis, which, in the long run, results in gut barrier dysfunction and low grade inflammation, a main trigger of CMD. The metabolism of dietary constituents by GM can result in protective or deleterious consequences for CM homeostasis including (i) fermentation of dietary fibers to generate the beneficial SCFAs, (ii) regulation of BAs metabolism with reduced cholesterol level and improved gut barrier function via multiple receptor pathways, (iii) metabolism of choline or L-carnitine to induce the release of the pro-atherogenic TMAO, and (iv) generation of favorable AA metabolites (such as indolepropionic acid) or noxious ones (such as TMAVA, and PAGln). As a key modifiable factor able to modulate GM composition, dietary regimens represent useful tools to target the gut/heart axis. The guidance for healthy diet patterns, recently revised by the AHA, converges on a whole foods approach, rich in fresh vegetables, fruits, and whole grain with limited intake of red meat and processed foods.

One main challenge in planning efficient nutritional interventions deals with the high inter-person variability in the response to the same diet. While in research studies machine learning approaches proved effective to harnessing the potential of gut microbiome-informed personalized nutritional advises, the implementation of these strategies on large scale in humans is still cost prohibitive. Another important issue is the uncertainties on diet duration in order to produce permanent GM remodeling in humans [221]. Intermittent fasting (IF), including periodic fasting and time restricted feeding, has been suggested to influence the GM and the host circadian clock, with improved CM health [222]. In spite of encouraging data from animal models, the contradictory results obtained in human studies hamper the translation of IF into clinical practice and highlight the need for a better mechanistic understanding of the interindividual variability in response to diet [220]. Sex-dependent microbial composition and sex hormones/GM crosstalk is emerging as a further critical aspect for the regulation of CM homeostasis [223]. However, there is still scarcity of data on this subject, thereby warranting further investigation.

Collectively, more well-controlled, prospective, longitudinal clinical studies are necessary to obtain a better mechanistic understanding of the inter-individual variability in response to diet and to define personalized dietary interventions to treat CMD.
